# Immunomodulatory effects of a cell processing device to ameliorate dysregulated hyperinflammatory disease states

**DOI:** 10.1038/s41598-024-63121-9

**Published:** 2024-06-03

**Authors:** Angela J. Westover, H. David Humes, Christopher J. Pino

**Affiliations:** 1 Nephrology/Internal Medicine, University of Michigan, Ann Arbor, MI 48109 USA; 2 Innovative BioTherapies, Ann Arbor, MI 48108 USA

**Keywords:** Translational research, Biological therapy

## Abstract

Cell directed therapy is an evolving therapeutic approach to treat organ dysfunction arising from hyperinflammation and cytokine storm by processing immune cells in an extracorporeal circuit. To investigate the mechanism of action of the Selective Cytopheretic Device (SCD), in vitro blood circuits were utilized to interrogate several aspects of the immunomodulatory therapy. SCD immunomodulatory activity is due to its effects on circulating neutrophils and monocytes in a low ionized calcium (iCa, Ca^2+^) blood circuit. Activated neutrophils adhere to the SCD fibers and degranulate with release of the constituents of their exocytotic vesicles. Adhered neutrophils in the low iCa environment display characteristics of apoptotic senescence. These neutrophils are subsequently released and returned back to circulation, demonstrating a clear potential for in vivo feedback. For monocytes, SCD treatment results in the selective adhesion of more pro-inflammatory subsets of the circulating monocyte pool, as demonstrated by both cell surface markers and cytokine secretory rates. Once bound, over time a subset of monocytes are released from the membrane with a less inflammatory functional phenotype. Similar methods to interrogate mechanism in vitro have been used to preliminarily confirm comparable findings in vivo. Therefore, the progressive amelioration of circulating leukocyte activation and immunomodulation of excessive inflammation observed in SCD clinical trials to date is likely due to this continuous autologous leukocyte processing.

## Introduction

Dysregulated cells of the body’s immunologic system cause a large number of complex clinical disorders, including sepsis, autoimmune diseases, and metastatic cancer. A therapeutic approach to return dysregulated cells to homeostatic function may be a novel approach to treat these disorders. Cell directed therapy is an evolving therapeutic approach with potentially wide application from organ dysfunction with tissue engineering to cancer immunotherapy with genetic engineering^1,2^. This report details a new form of cell directed therapy to phenotypically alter circulating leukocytes of the innate immune system to immunomodulate acute and chronic hyperinflammatory states resulting in proven clinical benefit.

Infection or tissue injury rapidly activates the innate immunologic system, primarily circulating neutrophils (NE) and monocytes (MO), to combat infection and to initiate tissue repair and recovery^3^. If this process is severe, a systemic hyperinflammatory response may occur and, oftentimes, becomes excessive and dysregulated. This dysregulated state leads to neutrophil interactions with tissue microvasculature and results in capillary sludging with tissue hypoxia and neutrophil egress from capillaries into tissue with release of various degradative products and toxic injury^4^. This combination of systemic tissue ischemia and toxic degradative processes oftentimes progress to multiorgan failure (MOF) and poor clinical outcomes^3^. Increased cytokine loads, termed cytokine storm, may or may not be related in cases. There is no universally accepted, single definition of cytokine storm, rather it is an umbrella term covering several disorders of immune dysregulation where cytokine levels are elevated, but etiology may be unclear^5^. To treat related conditions, approaches have focused on reducing the soluble mediators of this excessive and dysregulated process. These approaches have targeted individual cytokines, such as tissue necrosis factor (TNF)-alpha, interleukin (IL)-6 and IL-1 alpha (a), with monoclonal antibodies and non-specific removal of cytokines with sorbent-based technologies^6,7^. These approaches have not demonstrated improvements in durable clinical outcomes^6,7^. A serendipitous discovery during a clinical trial using a cell-based therapy utilizing an extracorporeal bioartificial kidney with human renal tubule progenitor cells to treat patients with acute kidney injury and multiorgan failure (MOF) resulted in the development of a cell directed extracorporeal immunomodulatory therapy, called a selective cytopheretic device (SCD)^8,9^. Development of SCD technology has been reviewed by Pino et al., which includes mechanistic insights previously gleaned from preclinical and clinical testing^3^. This device has proven to be successful in the clinical treatment of a wide variety of hyperinflammatory conditions with an excellent safety profile^10–16^.

The SCD is comprised of a polycarbonate cylindrical housing containing biocompatible polysulfone hollow fiber membrane, with a total surface area that, depending on the therapeutic dose, ranges from 1.0 to 2.5 m^2^. Therapeutic administration is delivered via an extracorporeal continuous kidney replacement therapy (CKRT) blood circuit that incorporates a low-shear stress blood flow path around the bundled fibers, thereby promoting binding of circulating neutrophils and monocytes, that are in an activated state due to a systemic inflammatory disease process^16,17^. The sequestered, activated leukocytes (LE) are immunomodulated when exposed to a low ionized calcium (iCa, Ca^2+^) environment (0.2–0.4 mM) afforded by well-developed clinical protocols for regional citrate anticoagulation (RCA) of the blood passing through the SCD^18^ and released back to the systemic circulation. SCD reduces systemic inflammation in a number of disorders with acute and chronic hyperinflammation, including sepsis^8,10,14^, AKI^10,14^, acute respiratory distress syndrome (ARDS) and cytokine storm associated with COVID-19^15,16^, hemophagocytic lymphohistiocytosis (HLH)^11^, hemolytic uremic syndrome (HUS)^11^, alcoholic hepatitis, acute on chronic liver failure^17^, and cardiorenal syndrome^12^ with improvement in clinical outcomes. With the successful translation of this platform technology in the clinical arena we undertook a series of investigations to better characterize the manner in which the SCD modulates the proinflammatory activity of circulating neutrophils and monocytes of the innate immunologic system. This approach is an example of the potential power of cell based or cell directed therapies as well as an example of translational medicine from bench to bedside back to bench.

## Methods.

### Porcine blood evaluation of iCa levels on LE-SCD interactions

**Figure 1:**
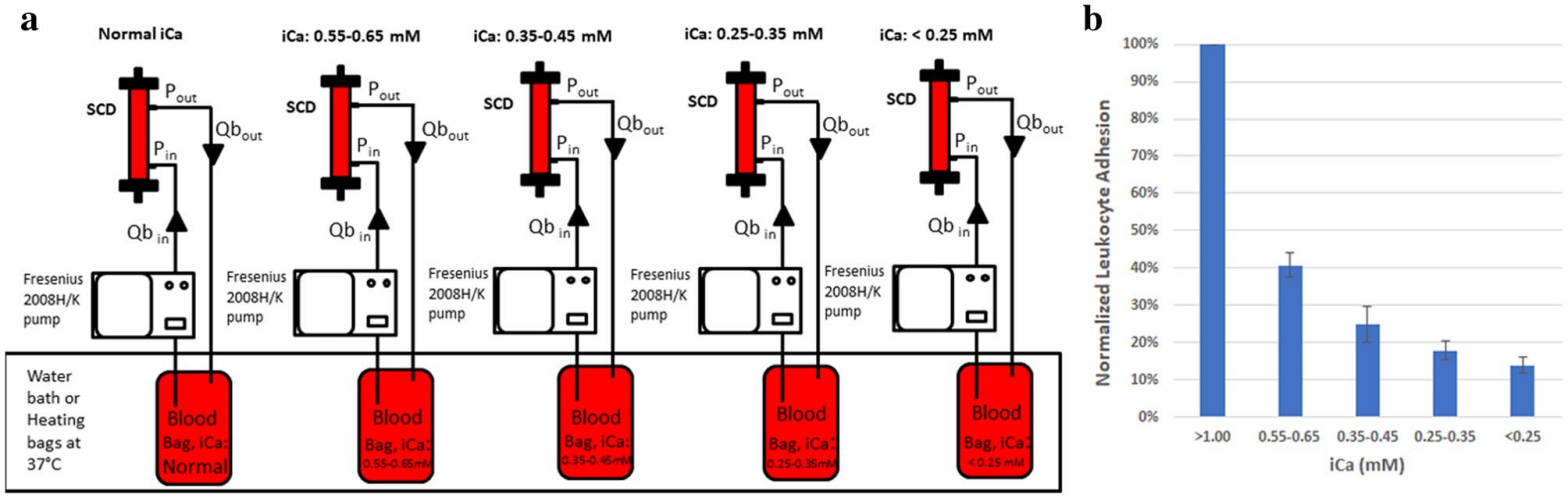
Circuit diagram for in vitro blood circuit (IVBC) studies (**a**). Reduction in ionized calcium (iCa) reduces leukocyte (LE) binding, without changing the differential representation of bound cells (**b**).

Porcine blood was obtained fresh from local abattoirs, where it was collected in 10U/mL heparin. Blood was transported back to the testing facility within 1 h, followed by filtration to remove clots and debris (SQ40S, Pall), 500 mL was pumped into each 1L EVA bag (Baxter) with 2 access ports, and citrate (ACD-A, Baxter) was added to reach prescribed ranges of iCa for testing. Blood was warmed to ~ 37 °C using dialysate warming bags (Phipps & Bird), and blood bags were gently mixed throughout study durations. Recirculating blood circuits were established by connecting Combi 8 mm blood segment dialysis tubing to the outlet and inlets of the blood bags, with the tubing adapted for SCD integration to allow for perfusion with Fresenius 2008H or 2008K dialysis pump systems. A representative diagram is shown in Fig. 1a. Pumps were utilized in service mode such that the blood pumps could be calibrated and utilized without requiring dialysate concentrate, as is the case with normal clinical operation. Circuits were initially primed with saline, before connecting blood bags. Normal saline (0.9% NaCl) was pumped single pass to waste, and recirculation was established once saline was removed. Blood flow rates for each circuit were set at the same 100 mL/min, a moderate blood flow setting for SCD therapy. After up to 4 h of perfusion, pumps were stopped, SCDs were disconnected from the circuits, and residual blood was rinsed out with normal saline, using at least 2× the fill volume of the SCD. Normal saline was drained and replaced with 0.2% EDTA in PBS at a pH of 7.2 which causes LE to dissociate from SCD fibers. SCDs were incubated for 30 min with elution buffer at room temperature followed by vigorous mixing and collection of the elution buffer filled with detached LE which were bound to SCD fibers. Total LE were assessed by fluorescent labeling of nucleated cells with Hoerscht 33342 and counted while viewed with a hemacytometer, or through the use of Leuko-TIC preparations (Bioanalytic GmbH). Differential counts of NE and MO were determined by Cytospin analysis. In brief, 50,000 cells were added to cytospin funnels in ~ 200 µL of buffer containing protein, and centrifuged at 800RPM for 3 min in a Shandon Cytospin centrifuge. Cells were deposited on a slide, stained using a differential stain kit (Newcomer Supply Part # 9112B) and counted using a microscope for NE and MO cell populations, with sets of 100 cells counted three times over and expressed as percentage of total cells.

### Human blood collection

Previous experience with in vitro blood studies suggested that fresh blood was required to study the molecular underpinnings of LE activation and SCD MoA, and that banked blood nor commercially-sourced human blood which must be drawn, tested and shipped, with > 24 h delivery times would not be representative of those processes. Fresh animal blood has been found to be adequate for preliminary evaluations, however due to better tools for the assessment of human disease including antibody availability, human blood is an ideal system for SCD MoA interrogation. However, full size, clinical SCD have a large blood priming volume, which would have necessitated very large volumes of blood to be drawn from donors. To limit the human blood draw volumes required, miniature SCDs and miniaturized circuits were utilized only requiring 50-100 mL total volume to be collected. Blood was collected directly into heparin, followed by addition of citrate (ACD-A, Baxter) to achieve an iCa of 0.25–0.4 mM. All experimental protocols related to human blood collection from donors were approved under the purview of the University of Michigan (UM) Institutional Review Board (IRB), HUM00209396. Human blood studies were carried out in accordance with associated guidelines and regulations. Informed consent was obtained from all participants.

### Construction of miniature SCD for use during in vitro human blood studies

**Figure 2:**
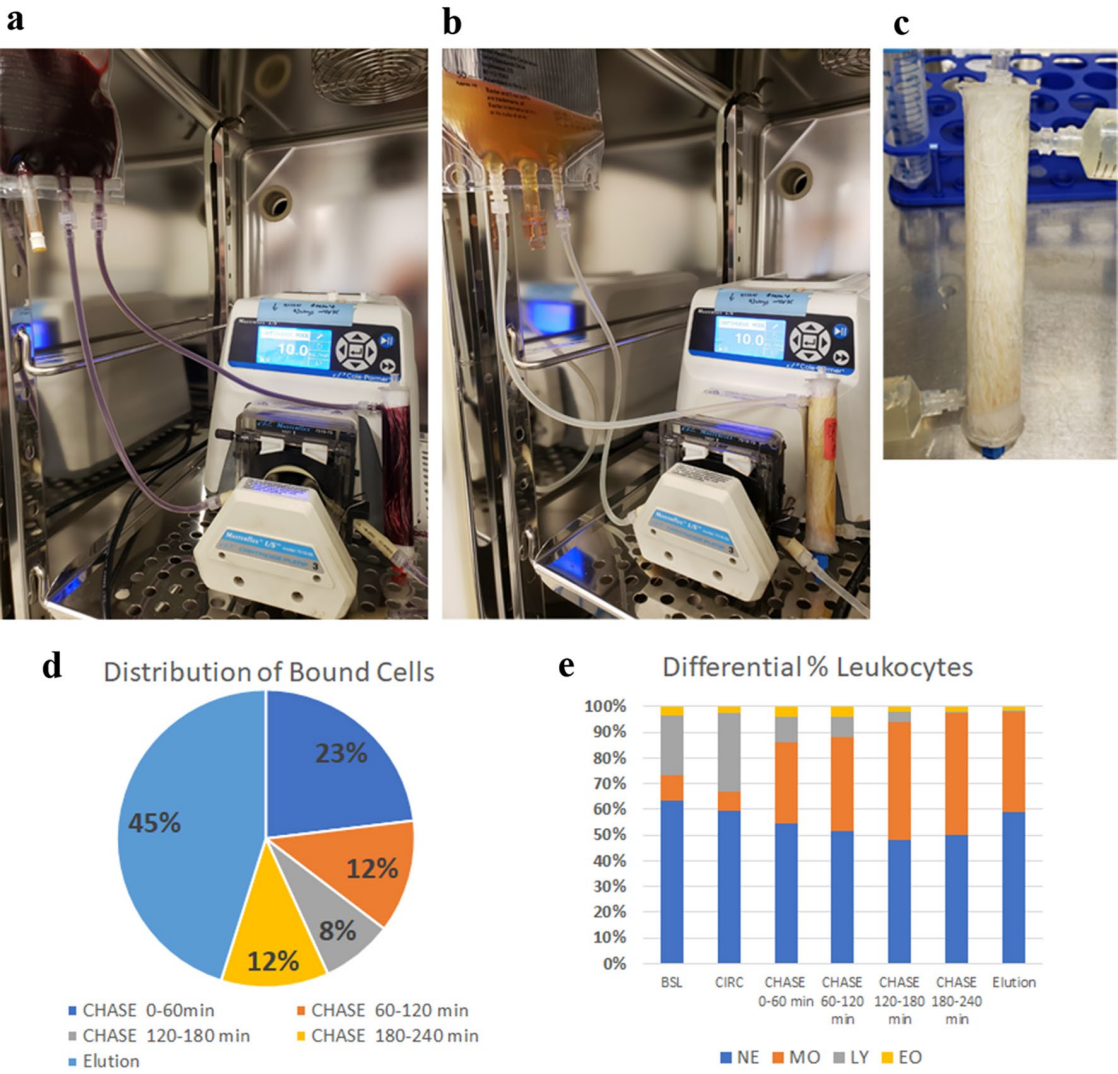
Photograph of miniature Selective Cytopheretic Device (SCD) being loaded with blood (**a**). The miniature SCD is rinsed with saline, and then perfused with plasma (**b**) for an additional 4 h period. Cells remaining at the end of plasma chase are then eluted to quantitate cells that remain adhered (**c**). A portion of bound cells that dissociate from the membrane during each chase period and final elution (**d**). The total number of leukocytes bound to the membrane at the end of blood circulation, prior to plasma chase, is set to 100%. The differential percentage leukocyte subsets in each step are shown (**e**).

Miniature SCD with an approximate fiber surface area of 0.1 m^2^ (Fig. 2a–c) were constructed using as similar materials as possible to the clinical devices. Fibers were harvested from clinical SCD (23 cm long) and placed within polycarbonate cases with an inner diameter of 1.9 cm (¾ in.) cut to a length of 13 cm. Ports for in and out blood flow were created by drilling the case 1 cm from each end, and inserting a female luer with threaded hex adapter, affixed using Dymax UV curing adhesive. Circular end caps were constructed from flat sheets of polycarbonate material cut to approximately 1inch diameter. These end caps were drilled in the center, and a female luer with threaded hex adapter was affixed with Dymax UV curing adhesive. One flat, circular end cap was affixed in place on one end of the cylindrical tube to initiate the potting process. Fibers within the cylindrical case were lifted such that polyurethane potting material could be applied through the female luer of the end cap. A fast-curing two-part polyurethane was used which was mixed up right before the potting process. The fibers bundles were pushed down into the potting material and spun in a swinging bucket centrifuge at 1300×*g* for 1.5 h to ensure that the potting material did not wick up the fibers while it cured fully affixing that end of the fibers. This process was repeated for the other side, with the fibers cut to length, end cap affixed, potting material applied, and centrifuged during curing. All materials used were heat stable and compatible with several forms of sterilization, allowing for sterilization of devices if required. Completed devices had a fill volume of ~ 20 mL. Miniature SCD circuits were comprised of ~ 1/8″ inner diameter silicone tubing, each with a BPT tubing segment (Fig. 2a,b), with a fill volume < 10 mL. All plastic parts for luer connections and barbed adapters were sourced from Nordson Medical.

### In vitro blood circuit (IVBC) with plasma chase with human blood

#### Blood recirculation period

Blood was recirculated for 2 h at a scaled-down blood flow rate (BFR) of 10 mL/min using a MasterFlex model 7523-80 with size 17 BPT tubing for the pump segment, along with silicone tubing with an inner diameter (ID) of 2.79 mm (Cole-Parmer) comprising a miniaturized circuit (Fig. 2a). The lower BFR mimics shear stresses of larger cartridges with greater cross-sectional areas. Samples were taken from baseline blood, and blood after 2 h of recirculation into ETDA sample vials. Blood samples were placed on ice until later for simultaneous batch processing with other samples. At the end of the recirculation period, the SCD and miniaturized SCD circuit were rinsed free of blood with normal saline.

#### Plasma chase period

Pooled human plasma generated by apheresis from heparinized blood was procured from Innovative Research (Novi, MI), and stored at − 80 °C up until use. Upon thaw, ~ 160–200 mL of pooled plasma stock was ultra-centrifuged to remove lipid aggregates and citrated to an iCa 0.25–0.4 mM (targeting 0.3 mM), before being split into to two separate stocks of 80-100 mL each. Plasma stock 1 was used in the plasma chase period 0–60 min after SCD LE loading (120–180 min in terms of total study time). After recirculating through the SCD for 1 h also at 10 mL/min (Fig. 2b), this plasma stock was entirely removed from the SCD circuit, split into two 50 mL conical tubes, and centrifuged at 500×*g* to collect SCD-released LE. Plasma stock 1 supernatant was removed and saved, leaving only a cell pellet for later analysis. Cell pellets were kept on ice, for simultaneous batch processing at the end of the study. Plasma stock 2 was used in the plasma chase period 60–120 min, with similar processing to stock 1, being centrifuged, plasma supernatant saved, and cell pellet collection. Plasma stock 1 was reused in the plasma chase period 120–180 min, and plasma stock 2 was reused in the plasma chase period 180–240 min, completing a total plasma chase of 4 h.

#### SCD elution and processing

As described for full sized cartridges above, to remove the remaining adhered leukocytes, an EDTA-based elution buffer was used to fill the device, which was incubated at room temperature for 30 min (Fig. 2c). Repetitive rinse steps with elution buffer were employed to remove remaining leukocytes. Previous analysis of this methodology has demonstrated that few residual cells not removed by elution process. Total elution volume was noted and split into conical tubes for centrifugation (500×*g*, 10 min) to isolate cell pellets, which were combined.

### Post-processing, CBC analysis, cell enumeration and antibody staining

Analysis of blood samples included complete blood counts (CBC) determined using an automated hematology analyzer (Drew Scientific Hemavet 950). Red blood cell (RBC) contamination in SCD elution and plasma chase cell pellets was removed by ammonium chloride lysis, at a ratio of 1 mL per 9 mL of lysis buffer. SCD-eluted cell number was enumerated by sub-sampling, staining with Hoechst dye (H33342) and direct fluorescence microscopy counts or trypan blue counts aided by a hemocytometer. All plasma chase cell pellets were enumerated by trypan blue counts. All cell samples were resuspended in 100 µl with 10^6^ cells, transferred to antibody tubes.

### Assay of neutrophil degranulation products

Representative primary (elastase), secondary (lactoferrin) and tertiary (MMP-9) granule proteins were measured in EDTA plasma using commercially available kits from Invitrogen (Lactoferrin EH309RB, Elastase BMS264, MMP-9 BMS2016-2). All samples, regardless of whole blood or plasma chase, were drawn into EDTA tubes, centrifuged at 1000×*g* for 10 min and cell free plasma stored at – 80 °C until assay. Baseline chase plasma levels were negligible. Because the same tranche of blood is used for Chases 1 and 2 and then chase 3, and 4, the net increase is reported for chase 3 and 4.

### Flow cytometric analysis of antibody panels

For cytometric analysis cells were adjusted to 10^6^ cells per test followed by 30-min incubation with fluorochrome-conjugated antibodies as shown in Table  1. Cells were fixed using BD FACSlyse buffer. Cytometric data was acquired with an Attune flow cytometer (ThermoFisher) and analyzed using FlowJo software. Panel compensations were auto generated by Attune software using lot matched antibody labeled ABC compensation control beads (ThermoFisher). Note live-dead marker was not used for all studies due to the observation of very few dead cells in the analysis and the ability to discriminate by autofluorescence.

**Table 1:** Antibody panels for Monocyte and Neutrophil flow cytometric analysis.

Systemic blood panel 1: Monocytes				
Target	Channel and label	Vendor	Catalog#	Clone
CD11b	BL1 FITC	BioRAD	MCA551F	ICRF44
CD91	BL2 PE	ThermoFisher	12-0919-42	A2MR-a2
CD14	BL3 PERCP Cy5.5^a^	BioRAD	MCA1568	Tuk4
CD192	RL1 Alexa Fluor® 647	BD Biosciences	558406	48607
CD16	RL3 APCH7	BD Biosciences	560195	3G8
CD197	VL1 BD OptiBuild™421	BioLegend	353208	Go43H7
HLA-DR	VL3 BD OptiBuild™605	BD Biosciences	562845	TU36

Systemic blood panel 2: Neutrophils				
Target	Channel	Vendor	Catalog#	Clone
CD11b	BL1 FITC	BioRAD	MCA551F	ICRF44
CD11b activated	BL2 PE	BioLegend	301406	CBRM1/5
CD10	BL3 PERCPCy5.5	BD Biosciences	563508	HI10a
CD62L	BL4 PE CY7	BD Biosciences	565535	DREG-56
CD33	RL2 Alexa Fluor® 700	BioLegend	303436	WM53
CD16	RL3 APCH7	BD Biosciences	560195	3G8
CD66b	VL1 BD OptiBuild™421	BD Biosciences	562940	G10F5
CD184	VL4 BD OptiBuild™711	BD Biosciences	740799	12G5

^a^Antibody conjugated using LNK142PERCPCY5.5 Kit (Bio-Rad).

BL: blue laser; RL: red laser; VL: violet laser.

## Results

### Porcine blood IVBC: impact of iCa

Multiple pre-clinical and clinical investigations have demonstrated the selectivity of the binding of circulating leukocytes on the SCD membranes. Neutrophils and monocytes but not lymphocytes are the dominant cell type that binds to the membranes^14,15,17^. Since the cell surface molecules responsible for binding of neutrophils and monocytes to endothelium and other surfaces are calcium dependent, we undertook studies to assess the effect of lowering of blood ionized calcium (iCa) utilizing an in vitro recirculating perfusion circuit on the binding kinetics of leukocytes in a SCD. As demonstrated in Fig. 1b, lowering of blood iCa from normal physiologic levels (1.0–1.2 mM) to less than 0.4 mM, had a progressive reduction in the absolute number of leukocytes bound within the SCD. All pairwise t-test comparisons with physiologic normal iCa demonstrated that all iCa reduction groups has significantly lower adhesion (p < 0.00011). Of importance, the distribution on cell type was not altered with the percentages of neutrophils, monocytes and lymphocytes similar at each iCa level, averaging for each iCa level at 90–93% and 3.9–4.1% for neutrophils and monocytes, respectively.

### Human blood IVBC

Previous results from preclinical and clinical studies have demonstrated that the SCD selectively adheres the more activated neutrophils (CD11b and CD66b) and pro-inflammatory monocytes (CD11b, CD14) from the circulation during a hyper-inflammatory state^15,17^. These cells express higher levels of CD11b, a non-discriminant adhesion molecule which generally represents a greater capacity for adhesion. Examination of the change of inflammatory phenotype of the released cells will provide an improved mechanistic understanding of the manner in which the SCD accomplishes this immunomodulation. In this regard, a simplified in vitro blood circuit (IVBC) model system was established and with the use of fresh human blood a comparison of the circulating, adherent and released LEs could be characterized. Following SCD adhesion of LEs during blood recirculation, the blood was rinsed out, and replaced with recirculating plasma to allow for subsequent analysis of released LEs, which were transiently sequestered, thereby evaluating the manner in which these cells may have been altered by the SCD.

### Leukocyte kinetics

The overall number of leukocytes in blood was estimated to be approximately 5 × 10^6^/mL. Therefore, approximately 5 × 10^8^ leukocytes were circulated through the miniature cartridge using 100 mL of blood. At the end of the circulation period (CIRC), around 10^7^ leukocytes, or 2% of the circulating leukocyte pool was associated with the membrane. Of these cells, as shown in Fig. 2d, approximately 60% of total bound cells were released in the next 4 h, and 40% remained membrane associated at the end of the chase period. Comparing the differential distribution of the BSL and CIRC blood to the bound cells revealed that monocytes and neutrophils have a high affinity for binding while lymphocytes do not. Of bound cells, approximately 60% were neutrophils and 30% were monocytes (Fig. 2e). The BSL blood was 10% monocytes, or 3 × 10^7^ total cells. Therefore, the 3 × 10^6^ monocytes total bound pool represents around 6% of the available pool at BSL. Similarly, the 1.35 × 10^7^ of the total bound neutrophils represented approximately 5.5% of the available pool at BSL. As presented, total bound cells refer to the number released over the 240 min of plasma chase and eluted from the SCD after 240 min of plasma perfusion.

### Neutrophil adhesion

**Figure 3:**
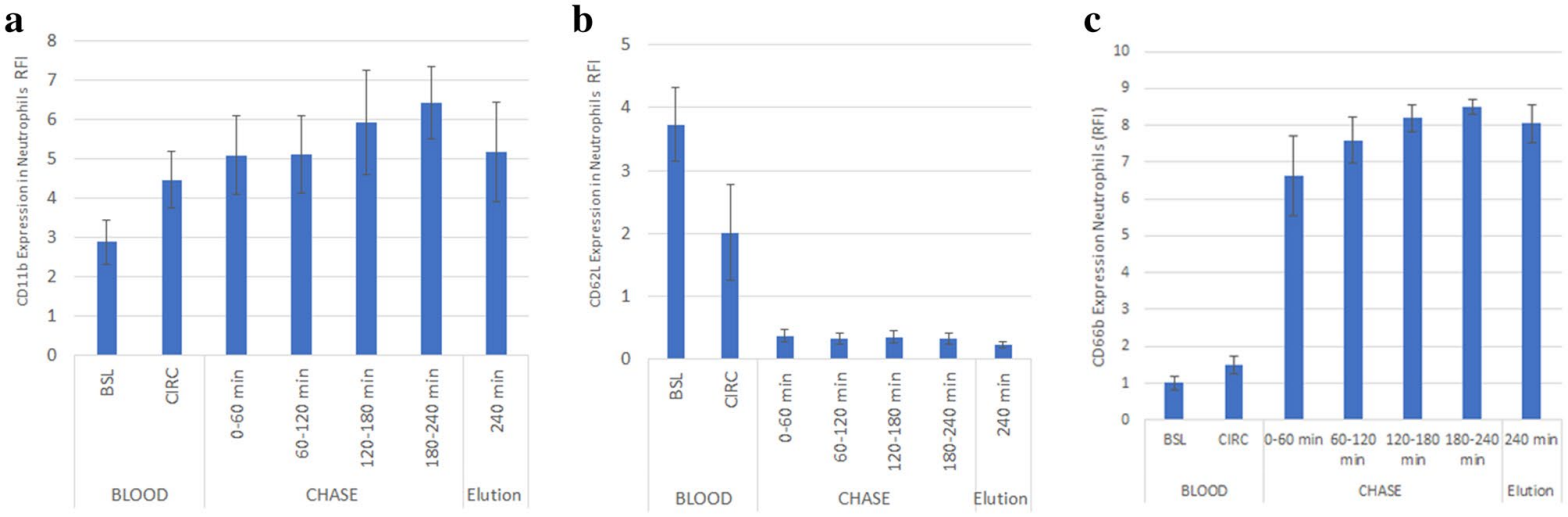
CD11b and CD62L expression of neutrophils populations derived from in vitro blood circuits. Increased expression of CD11b is associated with adhesion (**a**), reported as relative fluorescence intensity (RFI). Bound group had higher CD11b expression (p = 0.02). Shedding of l-Selectin (CD62L) in bound cells was highly significant in all bound groups compared to baseline (p < 0.01) (**b**). CD66b expression of neutrophils populations derived from in-vitro blood circuits, demonstrating release of CD66b upon binding to membrane (**c**).

Neutrophils can translocate integrins to the cell surface due to external stimuli to increase their overall ability to attach to surfaces, a phenomenon involved in the complex process referred to as neutrophil priming. CD11b is the alpha M component of complement receptor 3A representative beta Integrin. CD11b is a highly permissive integrin which binds to a wide range of ligands including several different components of extracellular matrix and intracellular adhesion molecules (ICAMs), and therefore is used as a surrogate marker to measure neutrophil priming and activation^19,20^. A significant increase (p < 0.03) in CD11b was observed in both CIRC and bound cells compared to BSL (Fig. 3a). l-selectin (CD62L) is involved in the initial tethering and rolling of neutrophils across surfaces and is shed upon arrest of a rolling neutrophil when a binding event occurs^21^. Circulating neutrophils have high L-selectin expression at baseline. Neutrophils released from the membrane during plasma chase and eluted at the end of the study had significantly lower L-selectin expression (p < 10^–10^), as seen in Fig. 3b. This reduction in CD62L supports strongly neutrophil binding to the membrane surface of the SCD. Neutrophils in CIRC compared to BSL had lower CD62L and higher CD11b expression, and may reflect some degree of cell binding and release during the first two hours of the study.

### Neutrophil activation

**Figure 4:**
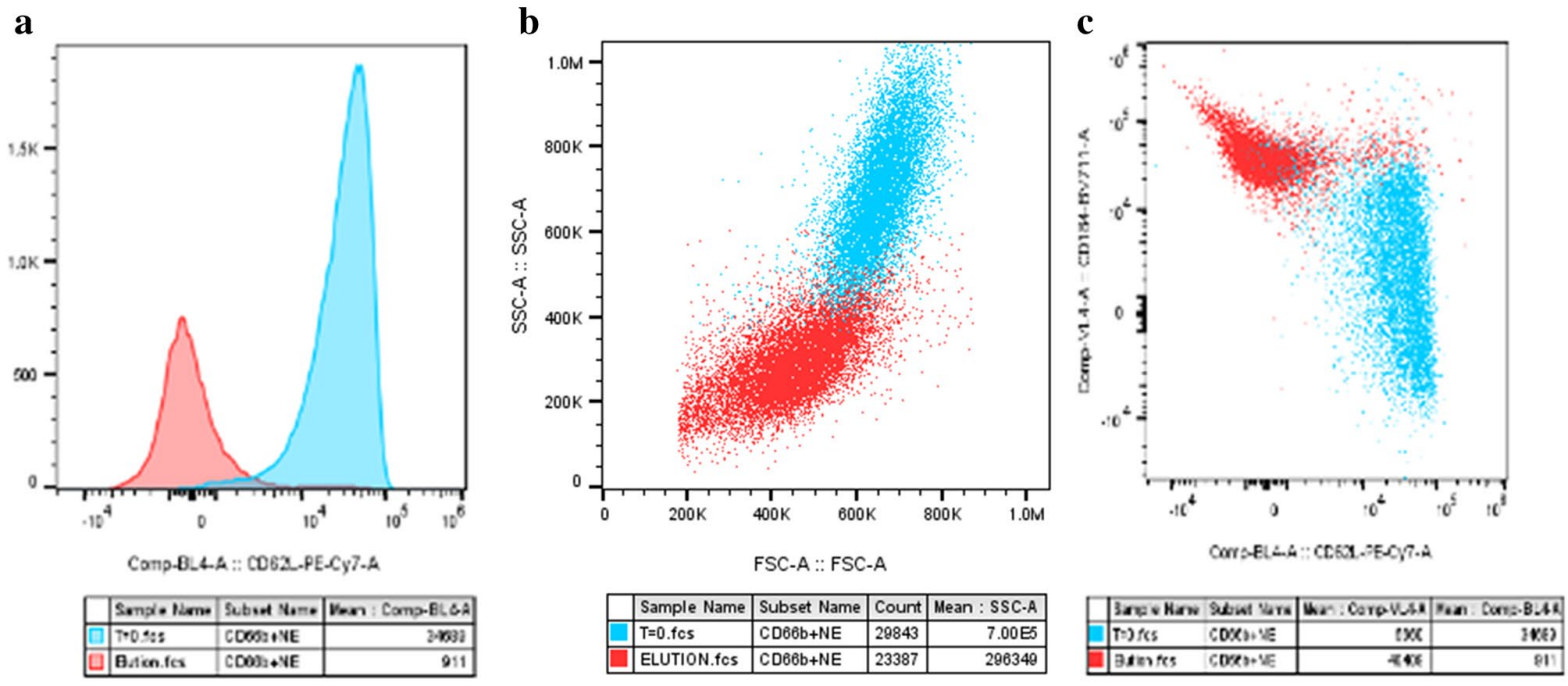
SCD released neutrophils exhibit degranulation and senescent phonotypes. Comparison of BSL blood (blue) vs. Eluted cells (red) shown. l-Selectin is shed from blood upon binding resulting in a left shift from high surface expression to low or no expression (**a**). Degranulation as measured by side-scatter area results in a shift down and to the left in scatter profile (**b**). The distinct change in phenotype between the two groups is shown when CD184 is plotted against L Selectin (**c**). Neutrophils lose l-selectin and gain CD184 resulting in an upward and left shift.

Neutrophil activation results in degranulation which can be measured by flow cytometry using the side scatter channel. Degranulated neutrophils fall in this channel because there are less granules to scatter the light. There was no significant change in neutrophil granularity BSL to CIRC. However, the bound cells were significantly degranulated compared to BSL and CIRC (p < 10^–6^ and p < 10^–5^ respectively). Upon neutrophil activation and degranulation, CD66b is rapidly released by secondary granules and consequently dramatically increases the surface expression of CD66b^22^. Accordingly, neutrophil cell surface expression of CD66b is a marker for activation and degranulation^22,23^. In this regard, the change in CD66b expression by bound cells was highly significant, p < 10^–10^ and P < 10^–5^ compared to BSL and CIRC respectively (Fig. 3c). The scatter profile of neutrophils is shown in Fig. 4.

**Figure 5:**
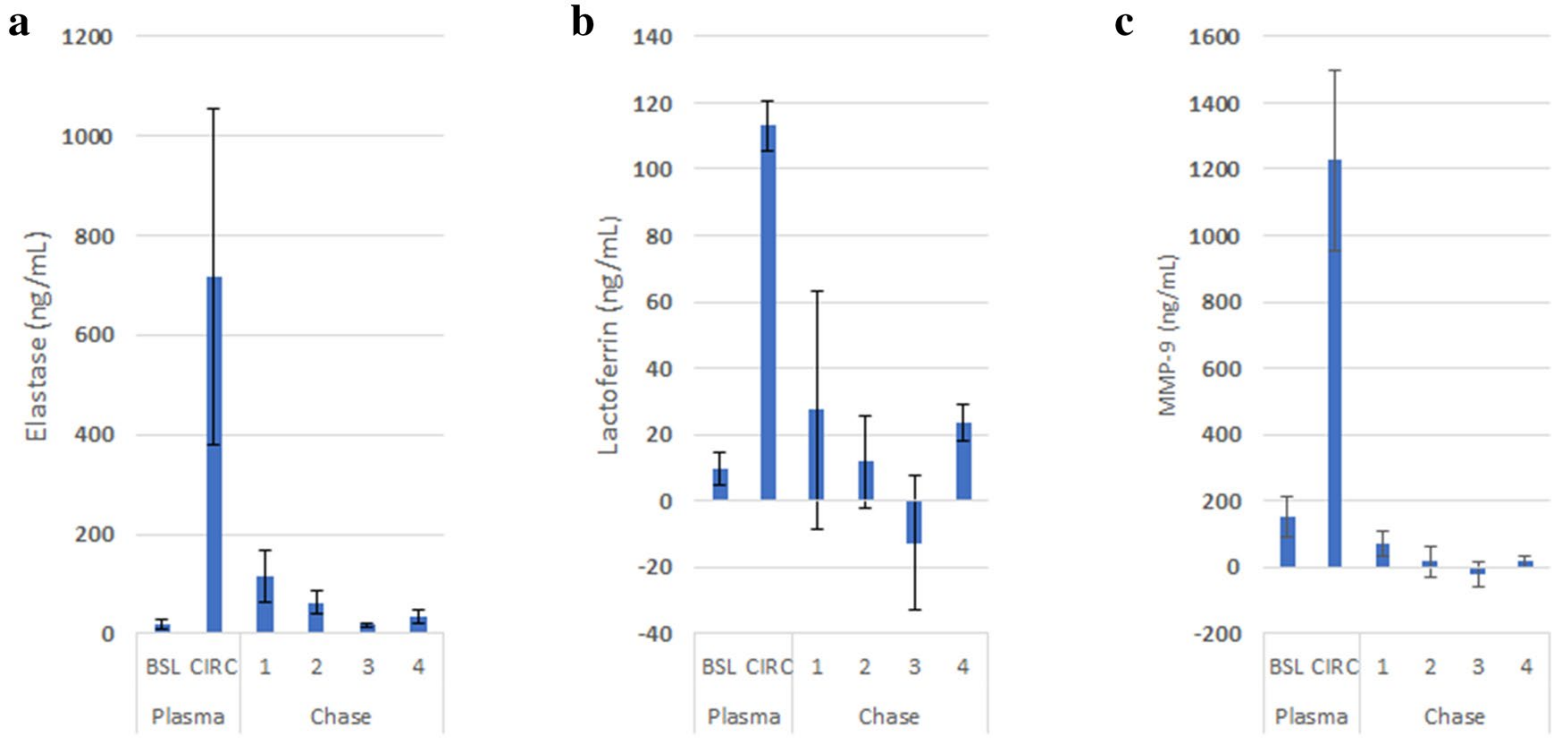
Contents of representative primary (elastase, **a**), secondary (lactoferrin, **b**) and tertiary (MMP-9, **c**) neutrophil granules are detected by ELISA in recirculated blood.

To confirm these events in this system, another set of three perfusion and chase experiments were done. As shown in Fig. 5, measurements of lactoferrin, elastase and MMP-9, as constituents of primary, secondary, and tertiary neutrophil granules, respectively, were measured during blood perfusion and plasma chase. A significant increase in plasma levels of these enzymes during the 120 min of blood perfusion was observed. No further degranulation occurred since no additional increases in levels were observed in the perfusate during the plasma chase experiments.

### Neutrophil senescence

**Figure 6:**
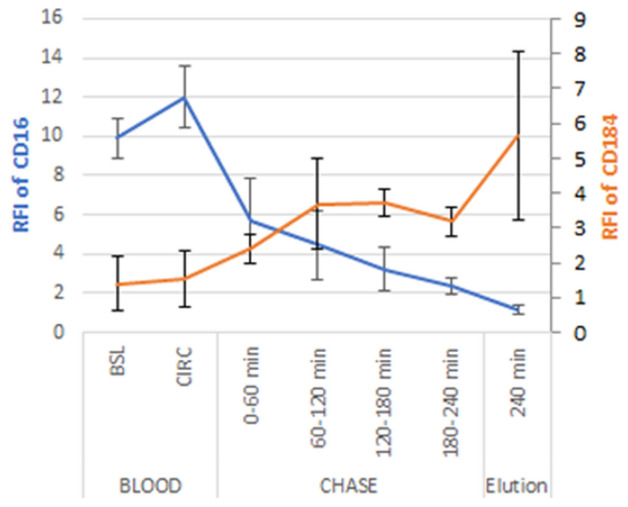
Relative fluorescence intensity (RFI) of CD16 and CD184 of neutrophils from in vitro blood circuits.

Primed or activated neutrophils have increased life-span and are resistant to apoptosis, while unstimulated neutrophils proceed to apoptosis in about 24 h. CD16−, CD184+ neutrophils are characteristic of the senescent apoptotic phenotype marked for clearance by bone marrow and other tissue specific macrophages^24,25^. CD184 is the C-X-C chemokine receptor type 4 and expression aids in the phagocytosis and digestion of apoptotic neutrophils for clearance from the circulation. CD16 expression was significantly reduced in bound cells compared to BSL and CIRC. Of note, CD16 expression decreased the longer cells were associated with the membrane while CD184 expression increased. For all studies, CD184 expression was higher in bound neutrophils compared to BSL and CIRC and highest in eluted cells (Fig. 6).

In summary, the data supports the following neutrophil events during blood perfusion in the SCD. Activated neutrophils bind to the SCD membranes and degranulate with release of the constituents of their primary, secondary and tertiary granules. Since declines in intracellular calcium promote the apoptotic program in neutrophils^26,27^, the low iCa environment due to citrate anticoagulation moves the bound cells to apoptotic senescence. Subsequent release of these leukocytes are returned to the circulation. Thus, during SCD treatment of a patient suffering from a systemic hyper-inflammatory state, this continuous autologous leukocyte processing of binding, degranulation, apoptosis, and release results in a progressive amelioration of circulating neutrophil activation and immunomodulation of the excessive inflammatory disorder.

### Monocyte phenotype distribution

The phenotypic classification of monocytes into subsets is complex and continues to be elucidated. Monocyte functional characteristics can vary within subsets defined as having the same surface markers. Still, monocytes can be generally sorted into three pools according to their cell surface expression of CD14 and CD16^28,29^. Thus, for these studies monocytes were categorized into classical (CD14+ CD16low), intermediate (CD14+ CD16+) or non-classical (CD14low CD16+) phenotypes. The classical and intermediate subsets have been shown to be pro-inflammatory while the non-classical phenotype is anti-inflammatory^30,31^.

**Figure 7:**
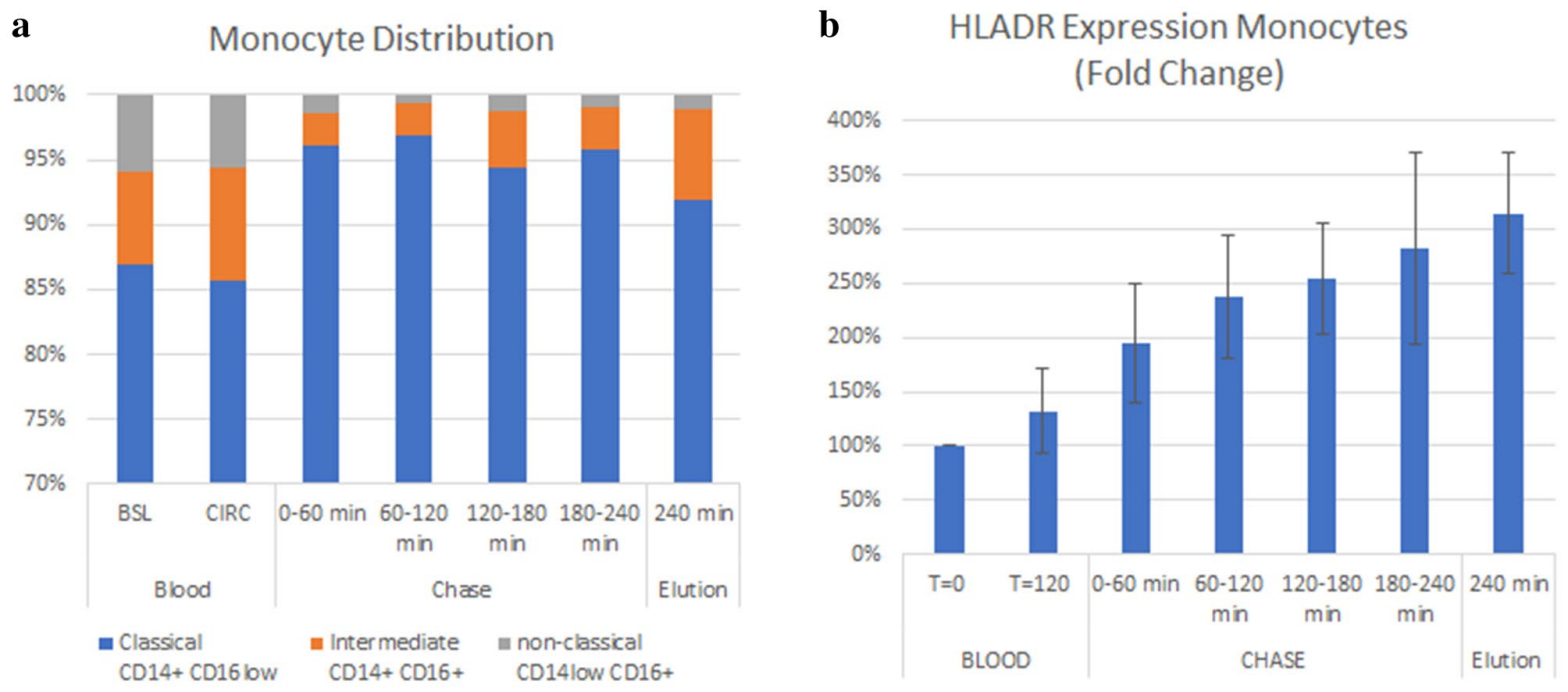
The distribution of monocyte subtypes (**a**) and HLADR expression (**b**) in circulating blood, plasma chase and elution phases of in vitro blood circuit studies.

Changes were not observed in the overall distribution of monocyte subtypes when comparing the BSL to the CIRC; however, the percentage of non-classical monocytes in the BSL (6.0%) and CIRC (5.6%) were significantly higher than in the bound cells (ranging from 0.7 to 1.4%, p < 0.05) indicating that few non-classical monocytes bound to the membrane (Fig. 7a). This difference is also reflected in a small but significant upward shift in the percentage of classical monocytes when comparing the BSL and CIRC blood to total bound cells (p = 0.019 and P = 0.002, respectively). These results demonstrate that the more pro-inflammatory subsets of the circulating monocyte pool are bound to the SCD but not the less inflammatory non-classical phenotype. HLADR expression was not significantly increased by monocytes that remained in circulating pool at 2 h (Fig. 7b). However bound cells had increased expression due to selection or stimulation that occurs upon binding in this system (Fig. 7b).

### Functional evaluation of monocytes

**Figure 8:**
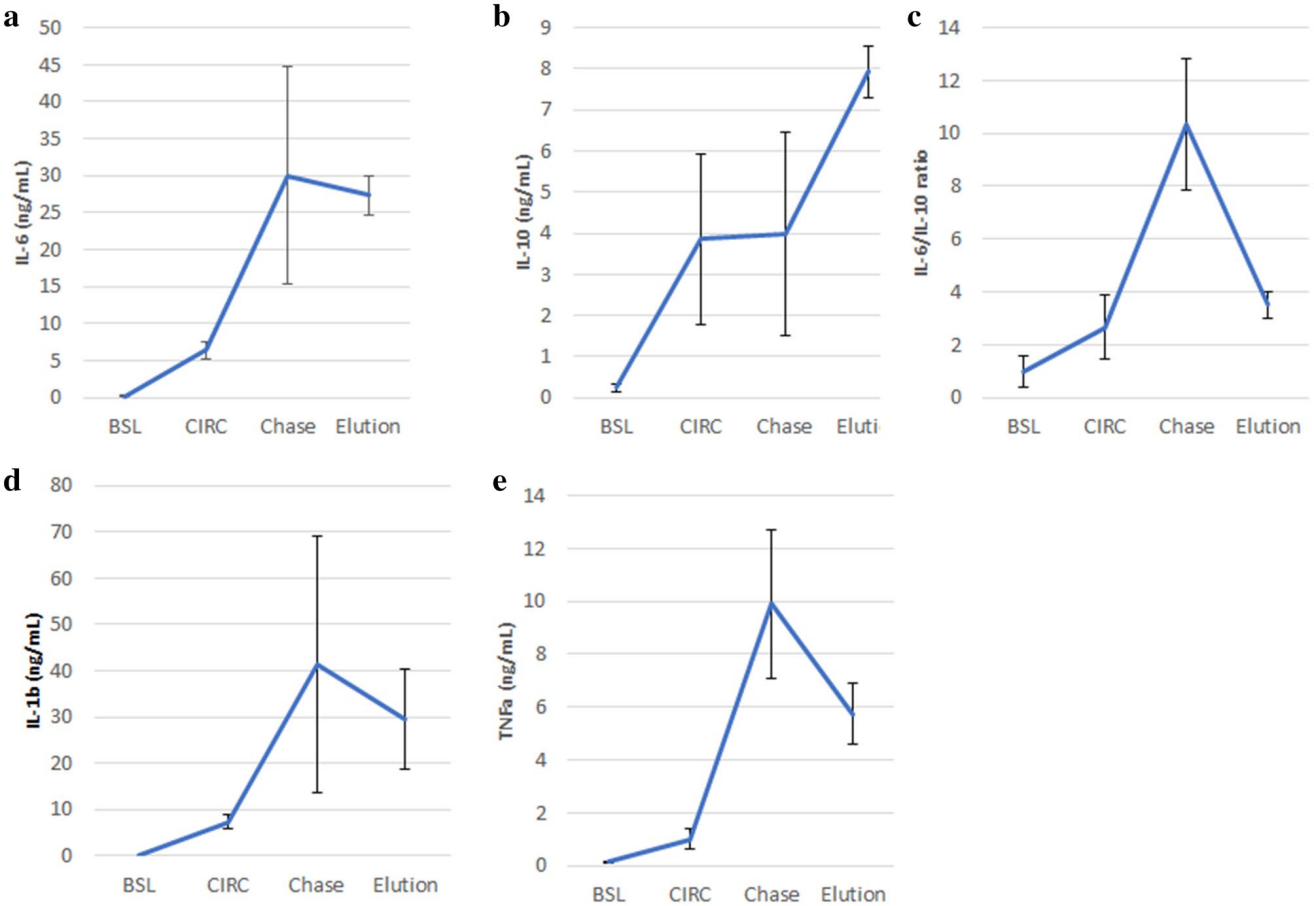
Monocytes isolated from in vitro blood circuits exhibit altered cytokine secretion due to SCD processing: IL-6 (**a**) IL-10 (**b**), IL-6/IL-10 ratio (**c**), IL-1b (**d**), TNF-α (**e**).

Since monocytes have substantial plasticity and the functional properties of the monocyte have yet to be clearly defined by expression of cell surface markers, additional studies in this system were undertaken to characterize the change in the functional activity, as assessed by measuring the secretion rate of key cytokines of circulating, bound and released monocytes^31^. In this regard, monocytes, enriched using Miltenyi pan monocyte isolation kit, were plated onto culture plates and incubated in cell culture media for 24 h. Supernatants were analyzed for levels of secreted cytokines, including TNF-a, IL-6, IL-10. The cytokine secretion rates of each sample (BSL, CIRC, Elution and Chase (Released Cells)) are diagramed in Fig. 8 and reported as ng/24 h/10^6^ cells.

As shown, monocyte activation occurs during the 2-h blood reperfusion with increases in cytokine secretion rates of all cytokines from BSL to CIRC. Further increases in cytokine secretory rates were seen in the eluted bound monocytes for IL-1b, TNF-a, IL-6, IL-6/IL-10 ratio compared to CIRC levels (Fig. 8), suggesting a change in monocyte functional phenotype towards a more inflammatory signature. Comparing the cytokine levels secreted from bound eluted cells versus released monocytes, declines in cytokine secretion was observed for TNF-a and importantly IL6/IL-10 ratios while IL-10 levels increased. The decline in IL-6/IL-10 ratio is especially important since decreases in this ratio are consistent with the release of less inflammatory monocytes from the bound monocyte pool^32^. This effect of the SCD to alter the phenotype of circulating monocytes from a pro-inflammatory to a less inflammatory subset has been observed clinically^13^.

In summary, the SCD with the low iCa environment results in the selective binding of more pro-inflammatory subsets of the circulating monocyte pool, as demonstrated by both cell surface markers and cytokine secretory rates. Once bound, over time a subset of monocytes are released from the membrane and this subset possesses a less inflammatory functional phenotype. This release results in an immunomodulatory effect on the circulating monocyte pool of patients with systemic inflammation.

## Discussion

SCD mechanism of action appears to be fairly complex and multifactorial, which has led to an evolving understanding as better toolkits and methodologies have become available. Mechanistic details that were discovered early in SCD development included that the SCD immunomodulatory effect appeared to require a blood flow path along biocompatible membrane surfaces with low shear stress approximating capillary shear and a low ionized calcium (iCa) environment (< 0.4 mM) promoted with regional citrate anticoagulation^8,14^. In fact, the shear stress (SS) of blood within a hollow fiber membrane in a standard hemofiltration cartridge was several-fold higher than arterial shear stress (> 30 dyne/cm^2^) compared to the shear stress along the outside surface of a hollow fiber membrane in the SCD below venular SS (< 1 dyne/cm^2^). Under these conditions the SCD membrane was hypothesized to selectively bind the most activated neutrophils and monocytes due to the calcium dependency of binding reactions of cell surface integrins on the leukocyte (1, 2 h). As demonstrated in Fig. 1, as the iCa level within the blood perfusion circuit decreased, the total amount of bound cells declined with the selective sequestration of neutrophils and monocytes. Further data, as summarized in Fig. 3, the bound leukocytes on the SCD membranes were more activated than those cells in the recirculating blood perfusate. The binding of neutrophils to the membrane surface of the SCD is also reflected by a significant decline in CD62L, also known as L-selectin, which is a cell surface ligand on neutrophils and is shed upon neutrophil binding to surfaces^21^. This selectivity of the SCD binding of highly activated neutrophils and monocytes and shedding of L-selectin has been seen in both preclinical animal models and clinical application of SCD therapy in various inflammatory disease states^8,12,15,17^.

Evaluation of the fate of leukocytes after binding, sequestration and release may provide insights into the mechanism by which the SCD influences the innate immune system. The results of these experiments demonstrate that upon binding, neutrophils degranulate with release of the constituents of exocytotic vesicles, including lactoferrin, elastase and MMP-9^30^. This process is also supported by increases in MFIs of the neutrophil cell surface marker CD66b. CD66b, or carcinoembryonic antigen, is a membrane constituent of neutrophil exocytotic vesicles and moves to the cell surface membrane upon degranulation^22,23^. An increase expression of CD66b is, therefore, seen upon neutrophil activation and degranulation events, as demonstrated in the released and eluted cells from the SCD membranes.

Prior work has demonstrated that at sites of inflammation neutrophil life span is extended due to delay in apoptosis^33^ and is, in large part, due to release of constituents of exocytotic vesicles^33^. Interestingly, bound and released neutrophils after degranulation in the SCD show a high degree of apoptosis, as reflected in a decline in CD16 and an increase in CD184 cell surface expressions. Decreases in CD16 cell surface expression has been well characterized with neutrophil apoptosis^25^. CD184, also known as CXCR4, is the receptor for stromal derived factor (SDF)-1, or CXCL12. The CXCR4/CXCL12 chemokine axis is critical in the trafficking of senescent/apoptotic neutrophils for clearance from the blood (26). The upregulation of CD184 cell surface expression of released and bound neutrophils were seen in these studies. This enhancement of neutrophil apoptosis and senescence with SCD interactions is likely due to the low iCa environment promoted by RCA within the SCD. Unlike other cells^34^, increases in calcium flux into the neutrophil delays apoptosis while declines in calcium entry promotes apoptosis^26,27^. These events are supported by an increase in percentage of released neutrophils expressing apoptosis markers. Subsequently, the released apoptotic neutrophils are phagocytosed and digested by the reticuloendothelial system and promotes a return to homeostatic immunologic balance. These events of neutrophil binding, degranulation and apoptotic progression have been observed in patients treated with the SCD.

The consequences of this neutrophil binding, deactivation and apoptotic progression within the SCD has been seen clinically. Elevated systemic absolute white counts (WBC) in a dysregulated immune cell response leading to cytokine storm, as typified in sepsis, occur in hyperinflammatory states due to delayed neutrophil apoptosis and cytokine stimulation of neutrophil release from bone marrow^24^. SCD treatment in patients with hyperinflammation due to multi-organ failure from AKI or COVID-19 results in significant declines in WBC^14,15^. Within hours after SCD therapy initiation, reductions in inflammatory cytokines are seen with a reduction in vasopressor requirements, enhanced respiratory function and ability to increase net volume removal with CKRT. These clinical improvements are most likely due to the amelioration of capillary leak and loss of intravascular volume to tissue interstitium from activated neutrophil and microvascular endothelium interactions^4^. This effect was clearly seen in a case series of pediatric patients suffering from HUS due to Shiga toxin E. Coli (STEC). This disease arises from directed abnormal leukocyte/endothelial interactions promoted by Shiga toxin that results in platelet activation and consumption, microvascular thrombosis, microangiopathic anemia, and organ tissue damage^11^. SCD treatment in these cases quickly reversed the progression of thrombocytopenia and anemia, most likely due to reversal of the pathophysiologic leukocyte/ endothelial interactions.

In a similar fashion, these results demonstrate that the most pro-inflammatory subsets of circulating monocytes (classical and intermediate) but not the anti-inflammatory reparative subset (non-classical) bind to the SCD in the low shear stress and low iCa environment. This selectivity of binding of more activated monocytes was shown with the functional assessment of the bound eluted pool of cells. The cytokine secretion rates of these sequestered cells demonstrated significantly higher rates of secretion of pro-inflammatory cytokines, TNF-a and IL-6, compared to circulating monocytes and a more inflammatory signature. Furthermore, the released monocytes during the plasma chase periods were shown to have a less inflammatory functional phenotype with lower rates of TNF-a and IL6/IL10 ratios secretion compared to bound cells. Because of the substantial plasticity of monocytes, characterization of the changes in monocyte activity promoted by the SCD will require more detailed evaluation. Further experiments are planned to assess the SCD related changes in the circulating, bound and released pools of monocytes with transcriptomic and metabolomic evaluation^35,36^.

This plasticity and complexity of SCD related changes in monocyte activity has been observed in recent clinical trials. SCD treatment in inflammatory disease states results in removal of the more inflammatory circulating monocytes using CD11b, CD14 and HLA-DR cell surface markers of activation^12,15,17^. The effect of SCD therapy in acute on chronic inflammatory disease state is especially noteworthy. An important role of pro-inflammatory intermediate monocytes (IM) in the progressive injury process in chronic inflammatory disorders, including chronic heart failure, cirrhosis and chronic kidney disease have been reported^28^. In these conditions circulating intermediate monocytes appear to migrate into previously damaged tissue and transform to pro-inflammatory macrophages (M1) to promote further degradative activity. The source of these intermediate monocytes appears to be in a splenic reservoir, which tend to be short-lived with half-life of 2 days or less^37^. In this regard, SCD treatment in patients with CHF and cirrhosis results initially in a reduction in circulating intermediate monocytes but two to three days later an increase in circulating IM occurs before returning to a normal distribution of the three phenotypes of circulating monocytes. The net effect of this therapy appears to deplete the splenic reservoir of IM and shift the circulating monocyte pool to a lesser inflammatory phenotype. This shift may result in improved tissue repair by rebalancing the tissue macrophage population in damaged tissue from a pro-inflammatory (M1) to a reparative/anti-inflammatory (M2) macrophage phenotype. This possibility may explain the effect of SCD treatment to reduce the progression to end stage renal disease after dialysis dependent AKI observed in several clinical trials evaluating SCD therapy in ICU patients with AKI and multi-organ failure^10,14^.

These experiments provide a better understanding of the mechanisms by which a cell directed therapy with continuous leukocyte processing in an extracorporeal circuit result in a clinical beneficial effect to immunomodulate, but not immunosuppress, complex dysregulated inflammatory processes. Similar methods to interrogate mechanism in vitro have been used to preliminarily confirm comparable findings ex vivo*,* utilizing a clinical SCD loaded with a patient’s leukocytes rinsed to be free of residual blood and chased with plasma to evaluate released, SCD processed LE, and LE that remain adhered to SCD at the end of plasma perfusion. Evaluation of neutrophil CD62L, CD66, and CD184, confirm L-selectin shedding, degranulation, and apoptotic progression (n = 1, data not shown). Additional studies are planned to confirm these preliminary observations. Further exploration of the manner in which the SCD influences the innate immunologic system in hyperinflammatory states may provide further understanding of the complexity of the host response to infection and tissue injury.

## Data Availability

As much as possible, data has been made available directly within the text, figures and legends of the manuscript. Additional data requests may be directed to the corresponding author.
